# Anaesthesia Concepts in Patients with Chronic Progressive External Ophthalmoplegia Undergoing Ophthalmic Surgery—A Retrospective Cohort Analysis

**DOI:** 10.3390/jcm13164710

**Published:** 2024-08-11

**Authors:** Nicolas Leister, Stefanie Wendt, Andrea Hedergott, Ludwig M. Heindl, Alexander C. Rokohl, Sandra E. Stoll, Erik Gordon, Bernd W. Böttiger, Julia Fricke, Volker C. Schick

**Affiliations:** 1 Department of Anaesthesiology and Intensive Care Medicine, Faculty of Medicine and University Hospital Cologne, University of Cologne, 50937 Köln, Germany; sandra.stoll@uk-koeln.de (S.E.S.); bernd.boettiger@uk-koeln.de (B.W.B.); volker.schick@uk-koeln.de (V.C.S.); 2 Department of Cardiothoracic Surgery and Intensive Care Medicine, Faculty of Medicine and University Hospital Cologne, University of Cologne, 50937 Köln, Germany; stefanie.wendt@uk-koeln.de; 3 Department of Ophthalmology, Faculty of Medicine and University Hospital Cologne, University of Cologne, 50937 Köln, Germany; andrea.hedergott@uk-koeln.de (A.H.); ludwig.heindl@uk-koeln.de (L.M.H.); alexander.rokohl@uk-koeln.de (A.C.R.); egordon.stud@gmail.com (E.G.); julia.fricke@uk-koeln.de (J.F.)

**Keywords:** rare disease, mitochondrial disorder, general anaesthesia, local anaesthesia, ophthalmic surgery

## Abstract

Background: Chronic progressive external ophthalmoplegia (CPEO) belongs to the group of mitochondrial encephalomyopathies. Anaesthesia for patients with CPEO may be associated with an increased risk due to known drug effects on mitochondrial metabolism. Therefore, the aim of this analysis was to evaluate anaesthesiological concepts in patients with CPEO requiring ophthalmic surgery. Methods: This is a retrospective, monocentric cohort analysis of eleven patients with CPEO undergoing ophthalmic surgery either with general anaesthesia or local anaesthesia in a German university hospital from January 2012 to February 2022. Results: A total of twelve ophthalmic surgery procedures were performed in eleven adult patients with CPEO. Six patients underwent surgery after receiving local anaesthesia (LA cohort). Five patients underwent six surgical procedures under general anaesthesia (GA cohort). In five cases within the GA cohort, propofol and remifentanil were used for the maintenance of anaesthesia. In one case, balanced anaesthesia with desflurane and remifentanil was used. The median duration of general anaesthesia was 37.5 min (range, 25–65 min). Patients stayed in the recovery room for a median of 48.5 min (range, 35–70 min). All patients were discharged on the first postoperative day. No relevant complications occurred in either the LA or GA cohort. Conclusion: Both local and general anaesthesia are feasible concepts for patients with CPEO undergoing ophthalmic surgery. Propofol, at least with a short duration (less than one hour) of use, appears to be a feasible hypnotic drug in CPEO patients.

## 1. Introduction

Chronic progressive external ophthalmoplegia (CPEO) causes ptosis and extraocular muscle paralysis, which are treatable with surgical intervention [1,2]. However, since CPEO belongs to the spectrum of mitochondrial encephalomyopathies, CPEO is associated with changes in ragged-red fibres, abnormal activity of cytochrome oxidase, and mitochondrial disorders, and plays a pivotal role in drug metabolism [3]. This mitochondrial disorder could result in impaired lipid metabolism and/or oxidative phosphorylation [4], thus resulting in a lack of adenosine triphosphate with a deficit in energy substrate storage [4,5,6]. Consequently, disturbances in the fragile metabolic balance must be avoided, which is particularly important in the context of surgical and anesthesiological procedures [5,6,7]. The current literature recommends that fasting for more than 4 h should be avoided (if necessary, bridged with a glucose infusion) and metabolic homeostasis should be monitored (through the measurement of lactate, glucose, and electrolytes) in mitochondrial disorder patients [4]. Indispensable ophthalmic surgical procedures are preferably performed through the application of local anaesthesia, but general anaesthesia may be considered necessary for patients with complex ophthalmic surgical situations (e.g., re-intervention) or relevant concomitant diseases, or as per patients’ requests. However, drugs used for general anaesthesia, especially propofol, may affect mitochondrial metabolism. Unfortunately, there is a gap in our knowledge about the metabolic effects of anaesthetic drugs in patients with mitochondrial encephalomyopathies; therefore, general anaesthesia may be associated with an increased risk for CPEO patients [8,9,10,11,12,13]. However, CPEO is a very rare disease, and the number of cases in the literature is limited. Existing descriptions of the anesthesiological procedures are at a case report level, but a consensus statement has provided guidance on the general anesthesiological management of patients with neuromuscular diseases since December 2022 [2,4,14,15,16,17]. Propofol appears to be a feasible induction drug, and its use for short-term total intravenous anaesthesia also appears possible, although its mechanism of action affects the metabolic processes in the mitochondria [4]. For this reason, other drugs (e.g., volatile anaesthetics, ketamine, opiates) are preferred [4]. Hypoglycaemic situations and hypo-/hyperthermia should be avoided [4]. The planning and management of surgical procedures should be individualised for each patient, as the risk profile can vary widely from patient to patient [4]. In particular, patients with Kearns–Sayre syndrome (KSS), a combination of CPEO with cardiac conduction disorders or cardiomyopathy, should be handled carefully, and the anaesthesiologist should be well prepared for the associated complications [14,18]. Patients with a mitochondrial disorder with a fragile metabolic state that can be affected by the chosen drug therapy, a lack of glycaemic substrate, or any other potential condition (hypo-/hyperthermia) [4,7,19]. Based on current knowledge, mitochondrial disease is not associated with malignant hyperthermia, but the risk of propofol infusion syndrome may be increased [4,20,21]. 

Prior to anaesthesia, CPEO patients should be carefully evaluated for general medical conditions, particularly cardiac comorbidities. The specific history of metabolic disorders, e.g., due to stress or during previous interventions/surgeries, should be obtained. Preoperative evaluation/preparation should be performed by experienced anaesthesiologists. 

With the trend towards outpatient care, especially for ophthalmological procedures, the question is whether this can be performed safely for CPEO patients in low-risk surgery. 

In order to improve patients’ safety, as well as the quality of the performance of the anaesthesia team caring for these patients, we retrospectively analysed all CPEO patients treated in our ophthalmologic surgery centre who underwent corrective surgery, with our primary objective being to compare patients with CPEO undergoing ophthalmic surgery with local anaesthesia (LA cohort) and patients with CPEO undergoing ophthalmic surgery with general anaesthesia (GA cohort). Moreover, we investigated the impact of using GA versus LA in patients with CPEO requiring surgery on their clinical outcomes (length of hospital stay, requirement of intensive care medicine) in detail (secondary objective).

The medical records of all adult CPEO patients undergoing ophthalmic surgery in our single quaternary centre (University Hospital of Cologne) were reviewed retrospectively. Patients were divided into two cohorts (GA and LA) and the relevant data on vital signs and the course of anaesthesia were obtained from the anaesthesia protocols. 

## 2. Materials and Methods

### 2.1. Ethics Committee Approval

This retrospective analysis was approved by the Ethics Committee (No: 21-1101) of the University Hospital of Cologne, Cologne, Germany (Chairperson Prof. Dr. R. Voltz) on 25 August 2022. The medical records of all patients with CPEO requiring ophthalmic surgery from January 2012 to February 2022 included in this study were reviewed. In accordance with hospital ethics, written informed consent was obtained for each medical procedure. The ethics committee waived the requirement to obtain informed consent from patients to review the data. This study was conducted in accordance with the Helsinki Declaration on Patient Safety in Anaesthesiology [22].

### 2.2. Patient and Data Selection

The medical records of all adult CPEO patients undergoing ophthalmic surgery in a single quaternary centre (University Hospital of Cologne) were reviewed. Patients were divided into two cohorts: patients with CPEO undergoing ophthalmic surgery with local anaesthesia (LA cohort) and patients with CPEO undergoing ophthalmic surgery with general anaesthesia (GA cohort). Thus, both cohorts were analysed regarding the length of hospital stay, the requirement of intensive care medicine, and relevant complications. The GA cohort was further analysed for the exact management of general anaesthesia (dosage, duration). 

The relevant data on vital signs and the course of anaesthesia were obtained from the anaesthesia protocols. The data were reviewed retrospectively and evaluated for this study by two independent examiners. 

### 2.3. Statistical Analysis

Continuous variables (age, weight, etc.) were compared using medians with ranges according to the data type and distribution. Categorical variables were compared using frequency counts and percentages.

The statistical analysis was carried out using SPSS version 29.0 (IBM, SPSS Statistics, IBM Corporation, Chicago, IL, USA).

## 3. Results

### 3.1. Demographic Data

Between January 2012 and February 2022, a total of twelve ophthalmic surgery procedures were performed in eleven adult patients with CPEO (Table 1). Six patients (two female and four male) underwent surgery with LA. The median age in this LA cohort was 42 years (range, 39–69 years). Six surgeries in five patients (two female and three male) were performed using GA. The median age in the GA cohort was 65.5 years (range, 32–79 years). The surgical procedures included strabism surgery and ptosis surgery.

### 3.2. Length of Hospital Stay

There was no significant difference regarding length of hospital stay upon comparing both groups of anaesthesia. Patients in the LA cohort as well as patients in the GA cohort were discharged on the first postoperative day, and thus, remained in hospital for 2 days. 

### 3.3. Performing General Anesthesia

Five patients underwent GA and one patient had two anaesthetic procedures with GA within the investigated timeframe.

In four anaesthetic procedures, patients were categorised into American Society of Anaesthesiologists (ASA) physical status classification group 3, and in two anaesthetic procedures, patients were classified into ASA group 2. Two patients were already diagnosed with KSS and were treated with an internal cardiac defibrillator/pacemaker. 

Intravenous induction of anaesthesia in all cases was performed with propofol (for dosage, see Table 2). Total intravenous anaesthesia (TIVA) with propofol and remifentanil was used for the maintenance of anaesthesia in five cases. In one case, balanced anaesthesia with desflurane and remifentanil was used (for details, see Table 2). The airway was secured using a laryngeal mask in all cases. For prophylaxis of postoperative nausea and vomiting (PONV), all patients received dexamethasone 4 mg and granisetron 1 mg. All patients received piritramide for postoperative analgesia, two patients in combination with metamizole and one with acetaminophen. The median duration of anaesthesia was 37.5 min (range, 25–65 min). Patients stayed in the recovery room for a median of 48.5 min (range, 35–70 min). 

In three cases, ptosis correction with frontalis slings surgery was performed, and in three cases, eye muscle surgery was performed.

### 3.4. Performing Local Anaesthesia

Six patients underwent surgery using LA: After topical anaesthesia with eye drops (oxybuprocaine 0.4%), 2–3 mL LA (prilocaine 2.0% and adrenaline) was injected into the upper lid with a 27-gauge needle. The operation was then started 1–2 min later. 

In one case, eye muscle surgery was performed with the topical application of oxybuprocaine and cocaine 4.0% and the subconjunctival application of 2 mL prilocaine 2.0% with adrenaline. 

In four cases, ptosis correction with frontalis sling surgery was performed, and in two cases, eye muscle surgery was performed.

In all cases, heart rate and oxygen saturation were monitored by the surgical team during the surgical procedure. All injections were performed by the surgeon. 

### 3.5. Short-Term Post-Anaesthetic Outcome

No relevant complications were recorded in either the LA or the GA cohort. All vital signs (e.g., blood pressure, oxygen saturation, heart rate, ventilation parameters) were consistently within the normal range for the patient’s age. All patients could be transferred to the normal ward and were discharged on the first postoperative day. In both groups, neither transfers to the intensive care unit nor readmissions after discharge were documented.

## 4. Discussion

### 4.1. Key Findings

In this monocentric, retrospective cohort analysis of a case series, CPEO patients undergoing ophthalmic surgery were treated successfully and without relevant clinical complications regarding whether anaesthesia was performed using LA or using GA.

### 4.2. Relationship to Previous Studies

To our knowledge, this is the analysis of a case series with the largest number of CPEO patients undergoing GA for ophthalmic procedures to date. Only case reports are available in the relevant literature. The 2022 consensus statement on anaesthesia in neuromuscular disease patients, published after our data collection, provided recommendations for perioperative anaesthesia management of this rare population. However, the use of certain medications (e.g., propofol) remains a case-by-case decision based on individual risk assessment [4,15,17,19]. 

All patients from the LA cohort in this analysis tolerated the surgical procedure well under LA, which is in accordance with the relevant literature [23,24]. However, there is also a case report with complications while using local anaesthetic in one patient [16].

Surprisingly, propofol was used for the induction of general anaesthesia in all analysed cases and for the maintenance of anaesthesia in five cases. Due to the known impairment of mitochondrial metabolism, propofol may be associated with metabolic side effects in these patients (e.g., risk of propofol infusion syndrome or mitochondrial metabolic disorder associated with increased mortality) [12,13]. However, with the dosage and duration used in our patients, anaesthetic drugs did not have a negative impact on patients’ clinical outcomes. This is consistent with the consensus statement, which considers propofol an induction drug and discusses the option of its short-term use in total intravenous anaesthesia [4]. There were no cardiopulmonary complications, either intraoperatively or postoperatively. Unfortunately, metabolic data (lactate, electrolytes, glycaemic profile) were not recorded. Our perioperative anaesthesia concept includes rapid postoperative mobilisation with the restoration of primary homeostasis (e.g., food intake, oral fluid replacement). No postoperative complications were documented, and none of the patients showed clinical signs of metabolic disturbance, so no further examinations were initiated or documented. All patients were easily discharged to the normal ward and were discharged the day after surgery without presenting any complications. 

Careful preoperative preparation should be performed by anaesthesiologists experienced in the management of patients with neuromuscular disorders. In addition to general medical conditions, cardiac comorbidities should be evaluated and a specific history of metabolic disorders, e.g., due to stress or previous interventions, should be obtained.

### 4.3. Weaknesses and Strengths

Our analysis has several limitations: The retrospective and single-centre nature of this analysis and the number of analysed patients limit the generalizability of the presented data. The limited number of patients included in this study reflects the low incidence of CPEO as a rare disease.

GA with propofol and remifentanil was administered safely in our cohort. It must be taken into account that the dosage and the short duration of anaesthesia were not sufficient to cause profound clinical disturbances among the assessed patients. All patients showed subjective well-being and normal vital signs. Due to the retrospective nature of this analysis, no additional data were collected for the sole purpose of the analysis. Therefore, no regular controls of serum lactate values or creatinine kinase or data on patients’ glycaemic profiles could be obtained. Clinical outcome data such as length of hospital stay and intensive care medicine requirement were recorded in addition to clinical treatment data and objective vital signs. Nevertheless, the short times required in the recovery room after GA, the non-necessity of treatment in an intensive or intermediate care unit setting, and the absence of readmissions after discharge reflects that short-term GA was well tolerated by these patients. 

### 4.4. Implications

According to these results and with increasing pressure to rationalise procedures in healthcare systems worldwide, it could be discussed whether an outpatient setting is feasible in ophthalmic surgical patients with CPEO, given the results of the present analysis. Prospective analyses with the peri-anaesthesia monitoring of metabolic values (lactate, electrolytes, glycaemic profile) should be performed to create a more reliable database. The use of propofol for prolonged periods or at higher concentrations requires special attention in future research. Until detailed analyses are available, healthcare teams should follow the recommendations in the consensus statement.

## 5. Conclusions

Both LA and GA appear to be feasible approaches in the management of CPEO patients undergoing ophthalmic surgery procedures. GA with propofol and remifentanil in this cohort did not lead to clinical complications when used for short-term anaesthesia. Patients could be discharged from the hospital without any problems. Propofol and remifentanil could be used safely for general anaesthesia in CPEO patients, at least for short durations of GA (less than one hour).

## Figures and Tables

**Table 1:** Patients’ demographic data.

	Cohort LA	Cohort GA
Patients; number (gender)	6 (2 female/4 male)	5 (2 female/3 male)
Age; years (range)	42 (39–69)	65.5 (32–79)

LA, local anaesthesia; GA, general anaesthesia.

**Table 2:** Drug doses for general anaesthesia.

Case	Induction Dose Propofol (mg/kg)	Maintenance	Maintenance Dose (mg/kg/h) or Expiratory Volume (%)	Remifentanil Dose (µg/kg/min)
1	2.7	propofol	4.0	0.3
2	2.8	propofol	4.9	0.2
3	2.0	propofol	6.5–8.0	0.3
4	2.2	desflurane	4.0	0.28
5	2.2	propofol	5.5	0.13–0.3
6	2.7	propofol	6.7	0.22

## Data Availability

The datasets used and/or analysed during the current study are available from the corresponding author on reasonable request.
